# Advances of optical miniscopes for *in vivo* imaging of neural activity in freely moving animals

**DOI:** 10.3389/fnins.2022.994079

**Published:** 2022-09-07

**Authors:** Kunpeng Chen, Zhaoshi Tian, Lingjie Kong

**Affiliations:** ^1^State Key Laboratory of Precision Measurement Technology and Instrument, Department of Precision Instrument, Tsinghua University, Beijing, China; ^2^Weiyang College, Tsinghua University, Beijing, China; ^3^Tsinghua-IDG/McGovern Institute for Brain Research, Tsinghua University, Beijing, China

**Keywords:** miniscope, fluorescence imaging, *in vivo*, multi-photon, real-time neural imaging

## Abstract

To study neural mechanisms of ethologically relevant behaviors including many social behaviors and navigations, optical miniscopes, which can be carried by the model animals, are indispensable. Recently, a variety of optical miniscopes have been developed to meet this urgent requirement, and successfully applied in the study of neural network activity in free-moving mice, rats, and bats, etc. Generally, miniature fluorescence microscopes can be classified into single-photon and multi-photon fluorescence miniscopes, considering their differences in imaging mechanisms and hardware setups. In this review, we introduce their fundamental principles and system structures, summarize technical advances, and discuss limitations and future trends, for *in vivo* imaging of neural activity in freely moving animals.

## Introduction

Optical microscopes play critical roles in current biomedical studies. So far, various imaging mechanisms have been proposed, in which the development of fluorescence labeling techniques has greatly promoted the fluorescence microscopy (FM). Benefiting from the advantages of specific labeling in FM, one can not only observe the structure and morphology of cells or organelles, but also study their functions at the cellular or subcellular scales. Especially after that the Nobel prize in Chemistry was awarded to the discovery of green fluorescence protein in 2008, various functional proteins have been developed for either optical imaging or optical manipulation *in vivo*. For example, the use of genetically encoded calcium indicators, such as GCaMP, enables the functional imaging of neural network activity *in vivo*, which greatly prompts the advance of neuroscience.

However, for *in vivo* imaging in freely moving animals, benchtop microscopes are generally too large and heavy to be carried by the model animals, such as mice, bats, etc. Thus, optical imaging is generally performed by fixing the heads of model animals under the microscope objectives. Apparently, this approach would not be suitable for studying the neural mechanisms of many social behaviors, such as caregiving, mating, and fighting (Zong et al., 2017; Chen et al., 2020). Therefore, the development of a miniature fluorescence microscope that can be carried by the model animals, enabling real-time observation of neural activity in freely moving animals, is of great significance in neuroscience research.

**FIGURE 1:**
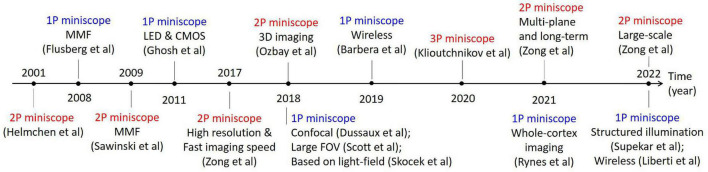
Typical works in the development of optical miniscopes for *in vivo* imaging in freely moving animals.

Recently, with the increasing integration of optical and mechanical components, and their decreasing masses and sizes, it becomes possible to fabricate miniaturized fluorescence microscopes for wearable applications. So far, a variety of optical miniscopes for free-moving animals, including mice, rats, and bats, have been developed, as shown in Figure 1. A miniscope is typically less than 5 g for mice, and a few tens of grams for rats. With miniscopes, one can perform *in vivo* observations of neural activity in freely moving states, for example, in studies of memory (Cai et al., 2016) and neurodegenerative diseases (Werner et al., 2019).

Based on the physical mechanisms of fluorescence excitations and the corresponding differences in imaging hardware, optical miniscopes can be classified into miniature single-photon fluorescence microscopes and miniature multi-photon fluorescence microscopes. The former is based on linear absorption effect and wide-field imaging, which has advantages in imaging speed as a result of parallel acquisition. However, it is susceptible to tissue scattering, which introduces fluorescence background and limits penetration depths. The latter is based on nonlinear absorption effects and laser-scanning imaging, which has advantages in deeper penetration and 3D resolutions at the cost of imaging speed. Thus, their system implementations are quite different.

The first optical miniscope was a two-photon fluorescence microscope, reported in 2001 by Helmchen et al. (2001). Following that, single-photon fluorescence miniscopes with multimode fiber (MMF) for delivering excitation light were developed in 2008 (Engelbrecht et al., 2008). Later, various schemes of optical miniscopes have been proposed and successfully applied in the study of neuroscience. In this brief review, we introduce their fundamental principles and system structures, summarize their technical advances, and discuss their limitations and future trends.

## Miniature single-photon fluorescence microscopy

Miniature single-photon fluorescence microscopes are based on single-photon fluorescence absorption, in which excitation photons are absorbed by the fluorophores and emission photons of a longer wavelength are collected for imaging. The power of emission fluorescence is linear to the power of excitation light. Based on wide-field imaging, the imaging speed can be fast. Moreover, the system architecture is not so complex, which is beneficial for its wide adoption. However, the non-localized excitation in single-photon fluorescence microscopes and tissue scattering introduce strong fluorescence background, which severely limits its penetration depths. Thus, new techniques, such as structured illumination (Supekar et al., 2022), are proposed to overcome this disadvantage.

### Structure of a single-photon fluorescence miniscope

**FIGURE 2:**
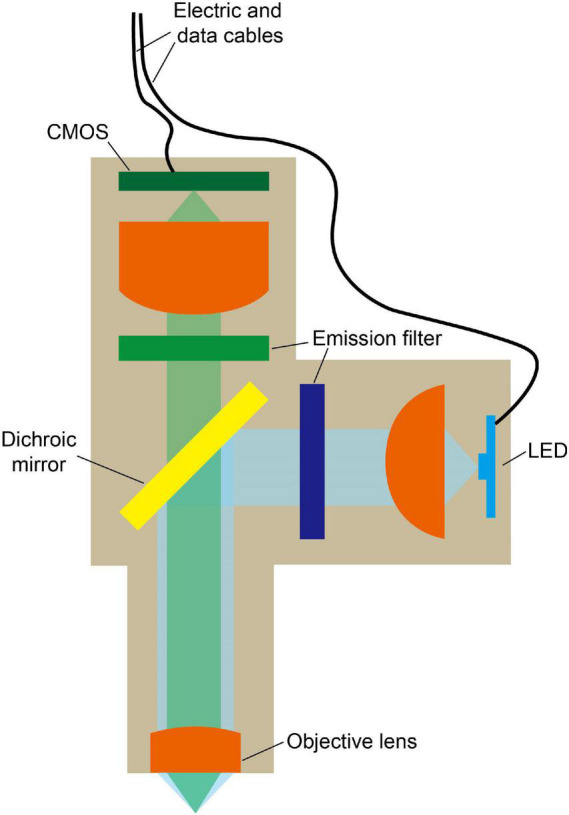
Typical model of miniature single-photon fluorescence microscope. LED is used for wide-field excitation and fluorescence signal is recorded by a CMOS. Dichroic mirror is used to separate excitation light from emitted fluorescence. Cables are used for power and data transmission.

The light path of a single-photon fluorescence miniscope is similar to that of a conventional fluorescence microscope, which includes an excitation path and an imaging path for exciting and collecting fluorescence, respectively. However, for portability, smaller and lighter components need to be selected. As shown in Figure 2, typical structures of a head-mounted, single-photon fluorescence miniscope (Ghosh et al., 2011; Scott et al., 2018; Barbera et al., 2019; Bagramyan et al., 2021; Rynes et al., 2021) include light source, filters, dichroic mirror (to separate excitation light from emitted fluorescence), objective lens, imaging sensor (such as CMOS camera), etc. In single-photon miniscopes, a high-brightness LED and a CMOS camera are usually integrated on the head-wearing part of the miniscopes for wide-field illumination and signal recording, respectively. Compared to the strategies using MMFs to deliver excitation light or using fiber bundle to export images in some early systems (Ferezou et al., 2006; Flusberg et al., 2008), this design is more integrated and stable. Meanwhile, MMFs are also used for special needs, such as replaceable multiple light sources (Srinivasan et al., 2019) and structure illumination based on coherent fiber bundles (Szabo et al., 2014; Supekar et al., 2022). Instead of using a complex microscope objective as in benchtop microscopes, a focusing lens or a GRIN lens is generally used. In which, GRIN lens can be attached in front of the targeted brain regions to perform *in vivo* imaging at deeper locations. However, it generally has limited NA, thus the excitation and collection efficiency are not high. So, in recent designs, a set of tube lenses are usually used in front of GRIN lens, which can improve NA and reduce optical aberrations. Besides, achromatic lens is also used to reduce chromatic aberration.

For power supply and data transmission, the single-photon fluorescence miniscopes are generally wired with electric and data cables (Ghosh et al., 2011; Scott et al., 2018; Bagramyan et al., 2021; Rynes et al., 2021). However, the cable, which connects the miniscope and the data acquisition module, increases the weight of the wearing parts, and the torque generated also interferes the animal activity. Zong et al. (2022) demonstrated that a heavy wearing parts (5 g) and thick cable (diameter = 1.5 mm) affects the mouse behavioral performance. Only when mice were equipped with a lighter wearing part (3 g) and a thinner cable (diameter = 0.7 mm), their behavioral performances became similar to that of free-moving mice (Zong et al., 2022). This indicates that the behavior of mice is sensitive to the weight of wearing parts.

### Performance of single-photon fluorescence miniscope

**FIGURE 3:**
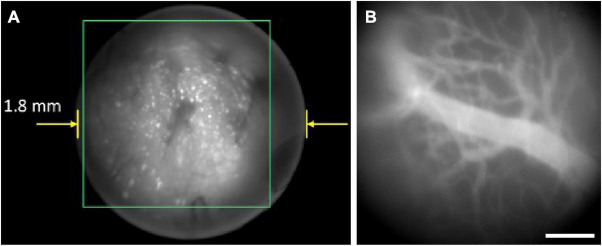
Typical results of single-photon miniscopes. Background fluorescence greatly influenced the imaging quality. **(A)** CA1 neurons in freely behaving rat with Miniscope-LFOV. Reproduced with permission from Guo et al. (2021). **(B)** Blood vasculatures stained with FITC at the surface of mouse cerebral cortex, recorded with UCLA Miniscope V4. Scale bar, 200 μm.

Benefiting from the parallel recording characteristics of wide-field imaging, single-photon fluorescence miniscope has the advantage of fast imaging speeds, with typical frame rates of tens of frames per second (fps) using CMOS on headpieces (Ghosh et al., 2011; Scott et al., 2018; Skocek et al., 2018; Aharoni and Hoogland, 2019; Liberti et al., 2022; Supekar et al., 2022), or several hundred of fps using fiber bundle to transfer images to record remotely (Ferezou et al., 2006; Flusberg et al., 2008; Dussaux et al., 2018). This enables the study of bio-dynamics over a large FOV, typically with a diameter of about several hundred microns to a few millimeters. However, same as conventional single-photon fluorescence microscopes, it has poor axial resolution and is easily interfered by background fluorescence, resulting in a limited imaging depth (typically 150 μm) (Ghosh et al., 2011). Figure 3 shows a typical result of a single-photon miniscope for imaging CA1 neurons in freely moving rats (Figure 3A), as well as blood vasculatures stained with fluorescein isothiocyanate (FITC) at the surface of cerebral cortex in freely moving mice (Figure 3B). It can be seen that there is clear background fluorescence, which restrained the penetration depth.

In functional imaging of neural network dynamics *in vivo*, the disturbance introduced by blood absorption cannot be neglected, especially in large-scale cortical dynamics imaging in mice and rats with FOV reaching tens of square microns. Thus, a reference light should usually be employed. For example, when using GCaMP as the functional indicator of neural activities, a green LED, together with a blue LED for exciting GCaMP, is generally used to obtain reflectance measurements for hemodynamic correction (Scott et al., 2018; Rynes et al., 2021).

### Recent advances in single-photon fluorescence miniscope

To reduce the weight of miniscope bearing on the model animals, one can turn to the wireless strategy for power supply or data transmission. As CMOS is used for image recording, it is possible to remove the cable and use a battery for power supply. Even though the battery increases the weight of the system, it can be carried on the back, instead of the head of the animal, reducing the impact of system weight on free movements (Barbera et al., 2019). The imaging data can be stored locally with a SD card, which can continuously collect data for about an hour, but this device is not capable of real-time observation. Recently, Liberti et al. (2022) reported their miniscope in freely flying bats with a wireless transmitter for real-time observation. The system is powered by a 3.7 V, 300 mAh lithium polymer battery, and the video is broadcast at approximately 2.37 GHz through a wireless transmitter (TX24019, 100 mW) (Liberti et al., 2022).

To get images with various fluorescence labels, a light source of multiple wavelengths should be adopted. However, compared to benchtop microscopes, the excitation wavelengths of light sources in the single-photon fluorescence miniscope are quite limited. Srinivasan et al. (2019) showed a miniscope with an integrated optical connector on the headpiece. Light from two lasers for both fluorescence excitation and photostimulation were combined into a MMF and delivered through the connector, which enabled simultaneous imaging and inhibiting of hippocampal neuron activity with jGCaMP7c and Jaws opsin, respectively. Thus, it is possible to study the causal relationships between neuronal network dynamics and animal behavior.

To study neural network dynamics across cortical regions, single-photon fluorescence miniscopes of large FOV are developed for *in vivo* imaging in freely moving rats and mice (Scott et al., 2018; Senarathna et al., 2019; Guo et al., 2021; Leman et al., 2021; Rynes et al., 2021). These miniscopes typically have FOVs of several tens of square millimeters, such as 7.8 × 4mm^2^ for rats (Scott et al., 2018) and 8 × 10mm^2^ for mice (Rynes et al., 2021). With large FOVs, it becomes possible to record mesoscale calcium activity and to study neurovascular coupling. In single-photon miniscopes, the interferences from hemodynamics should also be noted. So, both blue and green LEDs are integrated for fluorescence excitation and reflectance measurements, respectively, for hemodynamic correction (Scott et al., 2018; Rynes et al., 2021). Besides, Senarathna et al. (2019) developed a multi-contrast miniscope with a fluorescence channel, an intrinsic optical signal channel, and a laser speckle contrast channel, based on three illumination sources.

To achieve 3D imaging, light field microscopy, a scanning-free 3D microscopic imaging method, can be combined with single-photon fluorescence miniscope. By placing a microlens array in the position of the image sensor in conventional miniscopes and recording the light field image correspondingly, images at different angles can be achieved and be used for restoration by 3D deconvolution. Skocek et al. (2018) presented such a light-field miniscope (MiniLFM), which is capable of capturing neural network activity within a volume of 700 × 600 × 360μm^3^ at 16 Hz in the hippocampus of freely moving mice, and can robustly identify neurons at a distance of 15 μm. Recently, Yanny et al. (2020) improved the lateral resolution further by placing a phase mask on the Fourier surface.

**FIGURE 4:**
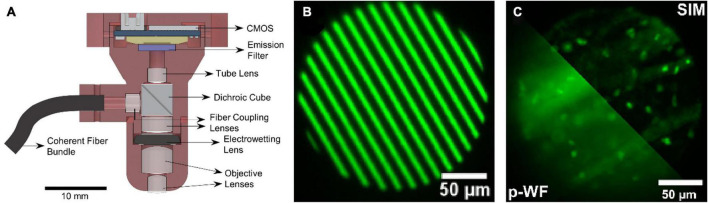
**(A)** Schematic of the SIMscope3D optical setup. **(B)** The spatial pattern generated on the digital micromirror device for structured illumination. **(C)** Structured illumination (SIM) reconstructed image (up-right), compared to the pseudo-widefield (p-WF) reconstruction image (down-left), in a fixed tissue sample of GFP labeling, showing the optical sectioning capability. Reproduced with permission from Supekar et al. (2022).

To inhibit the fluorescence background, structured illumination can be adopted in single-photon fluorescence miniscope, as in conventional wide-field fluorescence microscopes. Supekar et al. (2022) reported their miniscope (SIMscope3D) with electrowetting lens and onboard CMOS for high resolution volumetric imaging and structured illumination for the rejection of out-of-focus and scattered light, as shown in Figure 4. It delivers structured light using a coherent fiber bundle to obtain optical sectioning with an axial resolution of 18 μm. This is important for biodynamics studies, such as structure changes in supporting cells (including microglia) and immune cells.

## Miniature multi-photon fluorescence microscopy

Multi-photon fluorescence microscopy (MPM) was proposed to overcome the disadvantages of single-photon fluorescence microscopy, especially in penetration depth. Instead of absorbing one photon to reach the excited state, multiple photons should be absorbed simultaneously in MPM. Thus, the excitation wavelengths in MPM should be around twice longer, which reduces scattering and benefits deeper penetration. Moreover, based on the nonlinear effects of multi-photon absorption, the fluorescence excitation is localized around the focus, ensuring 3D resolutions. However, these advantages are achieved at the cost of complex systems.

### Design challenges of multi-photon fluorescence miniscope

The structure of multi-photon fluorescence miniscope is more complex than that of single-photon fluorescence miniscope in excitation, scanning, and detection. An interesting fact is that back in 2001, a two-photon fluorescence miniscope has already been reported (Helmchen et al., 2001), even earlier than most single-photon miniscopes. But it is not frequently used in biology experiments until the mature design emerged in recent years. In multi-photon fluorescence miniscope, the following difficulties should be overcome:

1. The delivery of excitation light to the objective. In multi-photon fluorescence miniscope, femtosecond lasers should be employed for fluorescence excitation. However, femtosecond lasers are generally bulky and heavy, making it difficult, if possible, to be installed as a headpiece. Instead, one can use optical fibers to deliver the laser to the objective. Unfortunately, the strong non-linear effects in optical fibers would degrade the quality of femtosecond pulses.
2. Laser scanning for 2D/3D imaging. As a laser scanning technique, beam steering should be performed in multi-photon fluorescence miniscope. Thus, miniature scanning devices are necessary to be installed in the headpiece.
3. Signal detection with a complicated point-detector. In multi-photon fluorescence miniscopes, fluorescence signals are detected with highly sensitive point-detectors, such as photomultipliers (PMTs). These detectors are generally too heavy to wear on animal heads.

**FIGURE 5:**
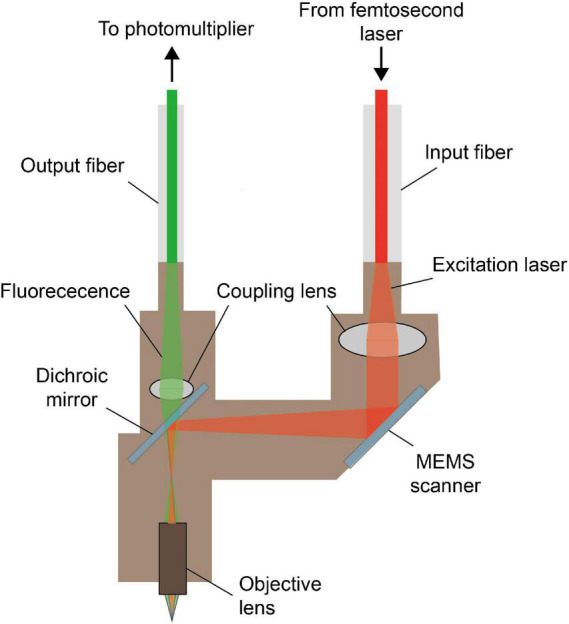
Typical model of miniature multi-photon fluorescence microscope. The excitation light from the femtosecond laser is delivered to the headpiece through the input fiber, and the fluorescence signal is collected and transmitted to the photomultiplier through the output fiber. MEMS scanner provides the transverse scan needed for multi-photon imaging.

So far, various schemes have been proposed and new components have been adopted, to improve the imaging performance of multi-photon fluorescence miniscopes continuously. As shown in Figure 5, different from that of single-photon fluorescence miniscope, typical structures of a head-mounted, multi-photon fluorescence miniscope do not include light source and imaging sensor, but include laser scanning hardware. The excitation femtosecond laser is generally delivered with optical fibers, and the fluorescence detection is usually performed remotely after collecting with optical fibers or fiber bundles.

In order to lessen the influence of non-linear effects during the transmission of femtosecond laser which highly influenced the quality of excitation light, custom designed hollow core photonic crystal fibers are adopted (Zong et al., 2017, 2022; Klioutchnikov et al., 2020). For example, a custom designed hollow core photonic crystal fiber HC-920 was employed, which transmitted hundreds of milliwatts of femtosecond laser pulses at 920 nm and the dispersion parameter is only ∼75 ps/nm/km (Zong et al., 2017). To minimize the dispersion broadening during pulse transmission in optical fiber, pre-compensation can also be performed, instead of adopting fibers with low dispersion. In the work by Ozbay et al. (2018), the laser is spectrally broadened through a polarization maintaining fiber and pre-chirped using a grating pair pulse stretcher. Li et al. (2021) use grating and prism to compensate for both material group velocity dispersion and third-order dispersion.

The laser scanning device increases the complexity of the headpieces in multi-photon fluorescence miniscopes. In the early version, scanning was performed by driving piezoelectric elements to vibrate the end of the fiber (Helmchen et al., 2001). However, it was rather slow. As the technology develops, smaller and faster scanning devices are adopted. Recently, MEMS (Zong et al., 2017, 2022; Klioutchnikov et al., 2020), a type of galvo, is used to scan at a high speed. As it is of small size, it can easily fit into the headpiece. Besides, Engelbrecht et al. (2008) showed that using miniature fiber scanner originally developed for video endoscopy as the scanning device is also viable. Instead, another solution is employing coherent fiber bundle (Ozbay et al., 2018) to deliver the scanning laser into the headpiece, in which the scanning is performed by a galvo remotely. This scheme avoids the problem of introducing scanning devices in the headpiece and can also lower the weight of the miniscope. But its spatial resolution is limited by the size of the fiber bundle.

In multi-photon fluorescence miniscope, the fluorescence signal is usually collected with optical fibers or fiber bundles, followed by sensitive detections with point-detectors, such as PMTs. Compared to the fibers that deliver excitation light, fibers for collecting signals are less demanding, as there is no necessity to maintain the shape of fluorescence signals. MMF is generally employed (Sawinski et al., 2009). Better fibers of high collection efficiency and the rotary joint could be employed to enhance optical and mechanical performance. Zong et al. (2017) designed a new type of supple fiber bundle, which has a higher collection efficiency than that of MMFs, for fluorescence transmission. They also used an electrical rotary joint between the PMT and the optical fiber to further reduce the obstruction to the mice’s movement (Zong et al., 2017). Li et al. (2021) designed an optoelectrical commutator to allow mice to rotate and walk freely, which provides excellent optical coupling stability (≤±1%) fluctuation during rotation and high torque sensitivity (< 8 mN ⋅ m). Their work shows that when optoelectrical commutator tracking was enabled, not only the mice behaved more actively, but also the cortex neurons exhibited increased firing activities (Li et al., 2021).

### Performance of multi-photon fluorescence miniscope

**FIGURE 6:**
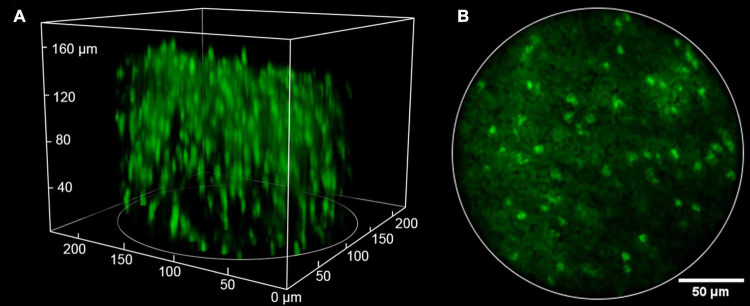
Typical imaging result of two-photon miniscopes. Reproduced with permission from Ozbay et al. (2018). **(A)** 3D volume of over 200 GFP-expressing oligodendrocyte cells (220 × 220 μm lateral × 180 μm axial). **(B)** An image from a single slice in the stack.

Compared to single-photon fluorescence microscopy, MPM has the advantages of deeper penetration, lower phototoxicity, and inherent 3D optical resolution, benefiting from the nonlinear excitation with near-infrared femtosecond lasers. These advantages preserve in multi-photon fluorescence miniscopes, as shown in Figure 6 (Ziv and Ghosh, 2015). Typical penetration depths below the surface in two-photon miniscopes can reach 250 μm (Zong et al., 2021). Meanwhile, laser scanning should be performed for 2D/3D imaging, which, unfortunately, limits the imaging speed. Recently, Zong et al. (2022) adopted a type of low Q-factor MEMS scanner and achieved 40 Hz frame rate at 256 × 256 pixels for FOVs around 410 × 410 μm^2^ to 420 × 420 μm^2^.

### Recent advances in multi-photon fluorescence miniscope

In addition to the advancements in the delivery of femtosecond pulses, the integration of novel laser scanning devices, and the collection of fluorescence signals, the capabilities in 3D imaging and deep penetration are further explored in multi-photon fluorescence miniscopes.

**FIGURE 7:**
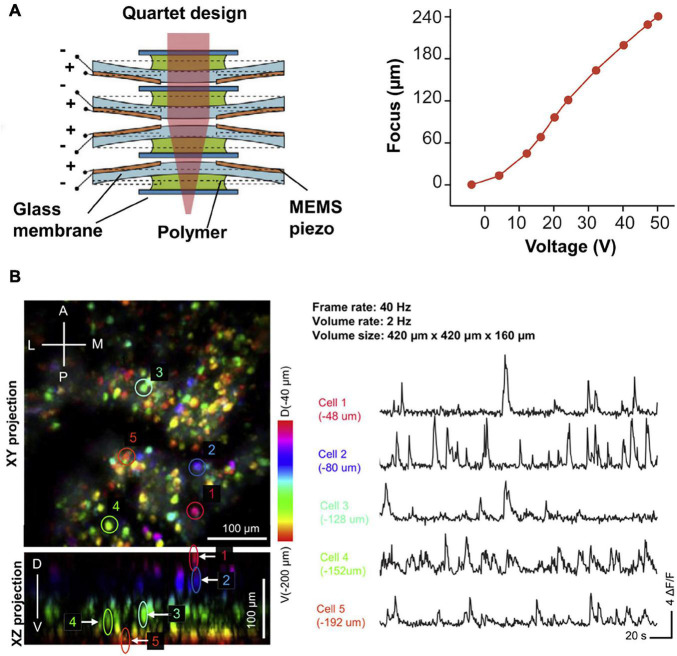
**(A)** Design and axial scanning range of μTlens (a nanotech micro-tunable lens), which is of only 0.06 g and has a response time of less than 0.4 ms. **(B)** 3D imaging of VC neurons in freely moving mice, at a volume rate of 2 Hz. Color indicates imaging depth. Reproduced with permission from Zong et al. (2022).

Taking the advantage in inherent optical sectioning, it is possible to perform 3D imaging with axial scanning elements, such as electrowetting tunable lens (EWL). As shown in Figure 6, Ozbay et al. (2018) reported a two-photon fluorescence miniscope with an EWL for multiple-plane imaging of neural activity. They demonstrated active axial focusing in 180 μm with an axial resolution of 10 μm. Additionally, joint control of axial and lateral scanning allows tilting of the focal plane, which provides a new capability in recording activities of functionally distinct neural layers. However, the frame rate only varied from 1.3 to 2.5 Hz in *in vivo* mouse imaging (Ozbay et al., 2018). Recently, some lighter axial scanning elements have also been adopted. Zong et al. (2022) used a nanotech micro-tunable lens (Farghaly et al., 2016), labeled as μTlens in Figure 7, which is of only 0.06 g and has a response time of less than 0.4 ms, for volumetric imaging. They demonstrated 3D imaging within a 420 × 420 × 160 μm^3^ volume, at a volume rate of 2 Hz (20 continuous planes), in which small calcium transients can be resolved.

To improve the penetration depth further, three-photon fluorescence microscopy (3PM) is developed, which achieves higher signal to background ratio (SBR) and thus breaks the depth limitation in two-photon imaging (Ouzounov et al., 2017). Considering that the effect of three-photon absorption is more localized, the background signal is much weaker and the SBR is much improved in 3PM. The same concept can be adopted in multi-photon fluorescence miniscopes, i.e., three-photon fluorescence miniscope can be developed with longer excitation wavelengths for deeper imaging. For example, Klioutchnikov et al. (2020) demonstrated a three-photon fluorescence miniscope for imaging neural activity in deep cortex of freely moving rats. With a hollow-core photonic bandgap crystal fiber operating at ∼1,320 nm for three-photon excitation of GCaMP6s, they showed the imaging of neurons and dendrites at a depth of up to 1,120 μm below the cortical surface (Klioutchnikov et al., 2020).

## Discussions and conclusion

Real-time monitoring of neuronal activity *in vivo* is critical in understanding the mechanism of neural network. Specifically for neuroscience studies which rely on freely moving rodents and bats, the development of lightweight and high performance fluorescence microscopes is of great significance. Thanks to recent developments in optoelectronic components, such as MEMS, GRIN lenses, EWL, fibers, etc., optical miniscopes of even better performances can be expected.

Moreover, the adoption of computational optics is of great potential to break physical limitations. For example, structured illumination and computational reconstruction can be adopted in single-photon fluorescence miniscopes to inhibit the fluorescence background. In multi-photon fluorescence miniscopes, strategies, including spatio-temporal multiplexing and point-spread-function engineering (Jin et al., 2020), can be adopted to improve the imaging speed and throughput, etc.

Except for monitoring brain activities, optical miniscopes can be also employed in the imaging of spinal cords of freely moving animals (Sekiguchi et al., 2016; Shekhtmeyster et al., 2021), which is indispensable in the study of motion diseases and motion disorders *in vivo*. In this case, the demanding requirement on the weight of optical miniscopes can be relaxed by a little.

In practice, accessibility issues with miniscopes have limited their broader application in neuroscience study. So far, a number of open-source single-photon miniscopes have been publicly available (Aharoni and Hoogland, 2019). However, components of multi-photon fluorescence miniscopes are still highly custom designed (Zong et al., 2022), which limits public accessibility. With more efforts paid to meet the urgent and developing needs, we can get deeper understanding of neural mechanisms of social behaviors and navigations which should be studied in freely moving animals.

## Author contributions

LK conceived the idea for this review topics and organization. KC and ZT performed the literature review under the guidance of LK. All authors contributed to the writing of the manuscript.
